#  Multifunctional lipid-based nanocarriers with antibacterial and anti‐inflammatory activities for treating MRSA bacteremia in mice

**DOI:** 10.1186/s12951-021-00789-5

**Published:** 2021-02-15

**Authors:** Chia-Chih Liao, Huang-Ping Yu, Shih-Chun Yang, Ahmed Alalaiwe, You-Shan Dai, Fu-Chao Liu, Jia-You Fang

**Affiliations:** 1 Department of Anesthesiology, Chang Gung Memorial Hospital, 5 Fuxing Street, Kweishan, Taoyuan, 333 Taiwan; 2 School of Medicine, College of Medicine, Chang Gung University, Kweishan, Taoyuan, Taiwan; 3 Department of Cosmetic Science, Providence University, Taichung, Taiwan; 4 Department of Pharmaceutics, College of Pharmacy, Prince Sattam Bin Abdulaziz University, Al Kharj, Saudi Arabia; 5 Pharmaceutics Laboratory, Graduate Institute of Natural Products, Chang Gung University, 259 Wen-Hwa 1st Road, Kweishan, Taoyuan, 333 Taiwan; 6 Research Center for Food and Cosmetic Safety and Research Center for Chinese Herbal Medicine, Chang Gung University of Science and Technology, Kweishan, Taoyuan, Taiwan

**Keywords:** Nanostructured lipid carriers, Ciprofloxacin, Rolipram, Bacteremia, Methicillin‐resistant *Staphylococcus aureus*, Sepsis

## Abstract

**Background:**

Bacteremia-induced sepsis is a leading cause of mortality in intensive care units. To control a bacterial infection, an immune response is required, but this response might contribute to organ failure. Kidneys are one of the main organs affected by bacteremia. Combination therapies with antibacterial and anti-inflammatory effects may be beneficial in treating bacteremia. This study aimed to develop nanostructured lipid carriers (NLCs) loaded with ciprofloxacin and rolipram that exert a combination of anti-methicillin-resistant *Staphylococcus aureus* (MRSA) and anti-inflammatory effects. Retinol was incorporated into the nanoparticles to transport retinol-binding protein 4 (RBP4) to the kidneys, which abundantly express RBP receptors. The NLCs were fabricated by high-shear homogenization and sonication, and neutrophils were used as a model to assess their anti-inflammatory effects. Mice were injected with MRSA to establish a model of bacteremia with organ injury.

**Results:**

The mean nanoparticle size and zeta potential of the NLCs were 171 nm and − 39 mV, respectively. Ciprofloxacin (0.05%, w/v) and rolipram (0.02%) achieved encapsulation percentages of 88% and 96%, respectively, in the nanosystems. The minimum bactericidal concentration of free ciprofloxacin against MRSA increased from 1.95 to 15.63 µg/ml when combined with rolipram, indicating a possible drug-drug interaction that reduced the antibacterial effect. Nanoparticle inclusion promoted the anti-MRSA activity of ciprofloxacin according to time-kill curves. The NLCs were found to be largely internalized into neutrophils and exhibited superior superoxide anion inhibition than free drugs. Retinol incorporation into the nanocarriers facilitated their efficient targeting to the kidneys. The NLCs significantly mitigated MRSA burden and elastase distribution in the organs of MRSA-infected animals, and the greatest inhibition was observed in the kidneys. Bacterial clearance and neutrophil infiltration suppression attenuated the bacteremia-induced cytokine overexpression, leading to an improvement in the survival rate from 22% to 67%.

**Conclusions:**

The dual role of our NLCs endowed them with greater efficacy in treating MRSA bacteremia than that of free drugs. 
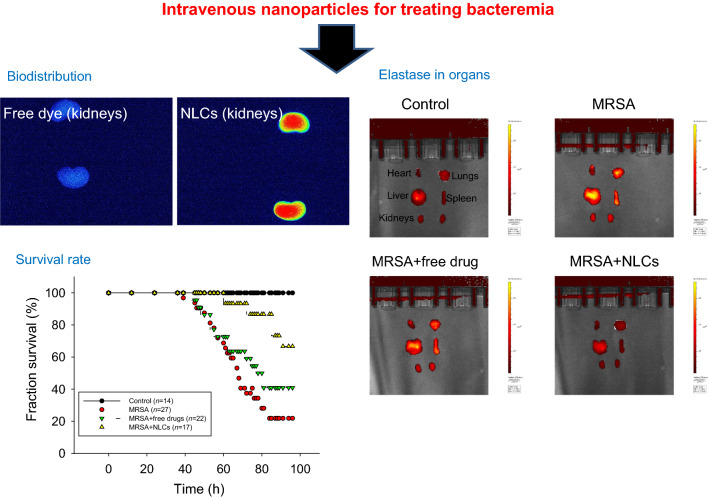

## Background

Bacteremia is described as the appearance of pathogenic bacteria in the bloodstream. The most common cause of bacteremia is *Staphylococcus aureus* infection [1]. In the United States, the annual incidence of *S. aureus* bacteremia has been 4.3−38.2 per 100,000 persons, with an associated mortality of 20%, since the 1990s [2]. Methicillin-resistant *S. aureus* (MRSA) is an additional bacterial burden with a high death rate. The mortality of patients with critical MRSA bacteremia can exceed 60% [3]. Individuals with bacteremia usually experience systemic inflammation due to a proinflammatory cytokine burst by immune cells [4]. Bacteremia, followed by acute inflammation, can lead to sepsis and multiple organ failure. Sepsis is an inflammatory and immune response to infection. The activation of proinflammatory mediators by sepsis contributes to organ damage, especially damage to the heart, lungs, and kidneys. The majority of bacteria identified in sepsis patients are gram-positive pathogens, such as *S. aureus*, with an increasing trend in drug-resistant microbes [5]. Severe septic shock is observed in 38%−40% of patients with *S. aureus* bacteremia [6].

Despite therapeutic innovation, the mortality rate due to bacteremia-induced sepsis is still high [1, 7]. Resistance to first-line antibiotics complicates the treatment of MRSA bacteremia [8]. The poor clinical outcomes highlight the urgent need for developing improved therapy for MRSA bacteremia. In the last decade, nano-based drug delivery systems have been increasingly used to improve antibacterial and anti-inflammatory treatments. The large surface-to-volume ratios and drug loading capacities of nanoparticles contribute to their biological advantages, namely, increased drug solubility, enhanced drug storage stability, improved bioavailability, prolonged half-life, and efficient organ targeting [9]. Nanoparticulate drug delivery is considered beneficial for the encapsulation of active agents against resistant bacteria and inflammatory disorders [10, 11]. Nanostructured lipid carriers (NLCs) are lipid-based nanocarriers that allow increased drug encapsulation because of the imperfect lipid matrix that is composed of lipids in both solid and liquid forms. The use of biodegradable lipids leads to higher tolerability of NLCs than that of polymeric and metallic nanoparticles [12]. There have has been no investigation on the application of nanomedicine to treat bacteremia. To date, clinical trials aiming to study anti-inflammatory approaches have failed to improve the outcomes of bacteremia-induced sepsis [13]; therefore, the development of novel therapeutic modalities with antibacterial and anti-inflammatory properties remains a critical issue. A new emerging regimen for inhibiting the inflammatory response is currently being investigated for the treatment of bacteremia-induced sepsis [7]. The use of treatments that combine antibiotics and anti-inflammatory therapies has been proven to reduce mortality in sepsis compared to the use of antibiotics alone [14, 15]. Therefore, this study explored the applicability of intravenous NLCs with combined anti-MRSA and anti-inflammatory activities for the treatment of bacteremia. To this end, ciprofloxacin and rolipram were both incorporated into NLCs.

The standard therapy for MRSA bacteremia includes the early intravenous administration of antibiotics, such as vancomycin, daptomycin, and teicoplanin [16]. Ciprofloxacin, a broad-spectrum antibiotic belonging to the class of fluoroquinolones, may also be intravenously administered to treat bacteremia [17–19]. To alleviate the inflammation stimulated by bacteremia or sepsis, phosphodiesterase 4 (PDE4) inhibitors have been proven effective in mitigating the cytokine storm induced by bacteria or viruses for the treatment of sepsis-induced organ damage [20–23]. The inhibition of PDE4 is associated with the suppression of immune overactivity via the prevention of cAMP degradation, as PDE4 can decrease cAMP expression to activate the inflammatory response. One representative PDE4 inhibitor is rolipram, which is a novel PDE4 inhibitor. Rolipram improves cardiac and renal function through the inhibition of cytokine overexpression in a rodent model of sepsis [24, 25]. Neutrophils are the most important cell in the host response to bacterial infection [26], and their migration is a fundamental component of the immune response elicited by bacteremia [12]. In this study, primary human neutrophils were used as model cells to examine the anti-inflammatory effect of NLCs. We established an MRSA-infected bacteremia model in rats to assess the inhibitory effect of NLCs on MRSA burden, acute inflammation, and mortality rate. We also monitored the biodistribution of the nanoparticles in various organs.

## Results

### Physicochemical features of NLCs

The particle size, polydispersity index (PDI), and surface charge are important features of nanocarriers that govern their stability and biological performance. As shown in Table 1, the mean diameter of the prepared lipid-based nanoparticles was 171 nm. The NLCs exhibited monodispersity with a PDI of 0.42. The zeta potential of the dual drug-loaded nanoparticles was highly negative at –39 mV. The encapsulation efficiency of the drugs determined by ultracentrifugation showed that the encapsulation percentages of ciprofloxacin and rolipram in the NLCs were 88.21 ± 7.0% and 95.81 ± 12.24%, respectively. The release of ciprofloxacin and rolipram from the nanocarriers was examined. The obtained profiles of ciprofloxacin release as a function of time indicated an initial burst release from the control vehicle and NLCs (the left panel of Additional file 1:  Figure S1). Ciprofloxacin release from the free control reached nearly 100% after 24 h, indicating a fast release. Inclusion in the nanoparticles decreased the release rate of ciprofloxacin. A similar trend was detected in the case of rolipram (the right panel of Additional file 1: Figure S1).

**Table 1:** Physiochemical properties of the nanostructured lipid carriers on nanoparticulate size, polydispersity index (PDI) and zeta potential

Property	Value
Size (nm)	171.40 ± 5.82
Polydispersity index	0.42 ± 0.04
Zeta potential (mV)	–38.80 ± 0.61

Each value represents the mean ± SEM (*n* = 3)

### Anti-MRSA activity of NLCs

**Fig. 1:**
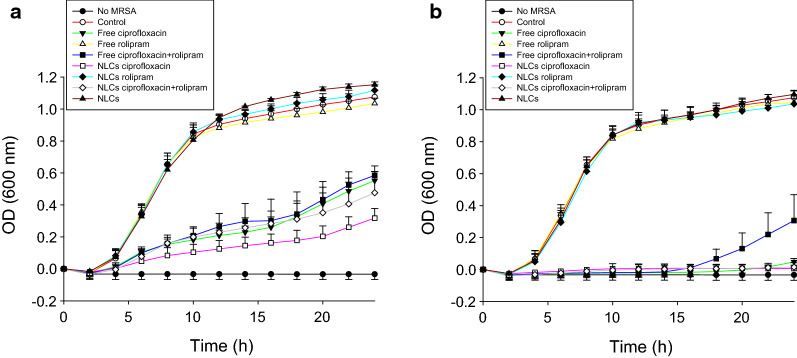
Determination of the antibacterial activity of free drugs and NLCs against planktonic drug-resistant MRSA:  **a **the time-killing curves of ciprofloxacin (0.5 µg/ml) and rolipram (0.2 µg/ml); and **b** the time-killing curves of ciprofloxacin (1 µg/ml) and rolipram (0.4 µg/ml). All data are expressed as the mean ± SEM (*n* = 3). MRSA, methicillin-resistant *Staphylococcus aureus*; OD, optical density; SEM, standard error of mean

The capability of the NLCs to inhibit MRSA growth was examined to understand their potential for treating MRSA-induced bacteremia. As shown in Table 2, the minimum bactericidal concentration (MBC) of ciprofloxacin alone was 0.98 µg/ml. Rolipram alone did not exhibit any anti-MRSA activity. Surprisingly, treatment with the combination of both drugs significantly reduced the anti-MRSA activity of ciprofloxacin. The MBC of ciprofloxacin increased by 8-fold after rolipram intervention. The antimicrobial potency of the NLCs loaded with ciprofloxacin was found to have a MBC between 0.98 and 1.95 µg/ml. Similar to their free forms, the combination of ciprofloxacin and rolipram in the NLCs increased the MBC of ciprofloxacin by 4−8-fold. Time-kill curves provide information regarding the extent and rate of anti-MRSA activity. Figure 1a and b show the time-response MRSA growth inhibition of ciprofloxacin and rolipram at low (0.5 and 0.2 µg/ml) and high doses (1 and 0.4 µg/ml), respectively. The control group was the MRSA population not treated with drugs or nanoparticles. Treatment with rolipram, in both its free and nanoparticulate forms, had no significant influence on MRSA growth compared to the no treatment control. The NLCs without drugs also showed no MRSA inhibition; however, the formulations containing ciprofloxacin markedly suppressed the growth of MRSA. The ciprofloxacin-loaded NLCs were more efficient than free ciprofloxacin in inhibiting bacterial growth. At high doses, free and nanoparticulate ciprofloxacin completely inhibited MRSA growth over 24 h. However, a combination of ciprofloxacin and rolipram in free form weakened the anti-MRSA effect.

**Table 2:** The MBC (µg/ml) of ciprofloxacin in free form and nanostructured lipid carriers in the presence or absence of rolipram against MRSA

Formulation	MBC (µg/ml)
Ciprofloxacin in free form	0.98
Ciprofloxacin + rolipram	7.81
Ciprofloxacin in NLCs	0.98‒1.95
Ciprofloxacin + rolipram in NLCs	7.81

Each value represents the mean ± SEM (*n* = 3)

### Anti‐inflammatory activity of NLCs

**Fig. 2:**
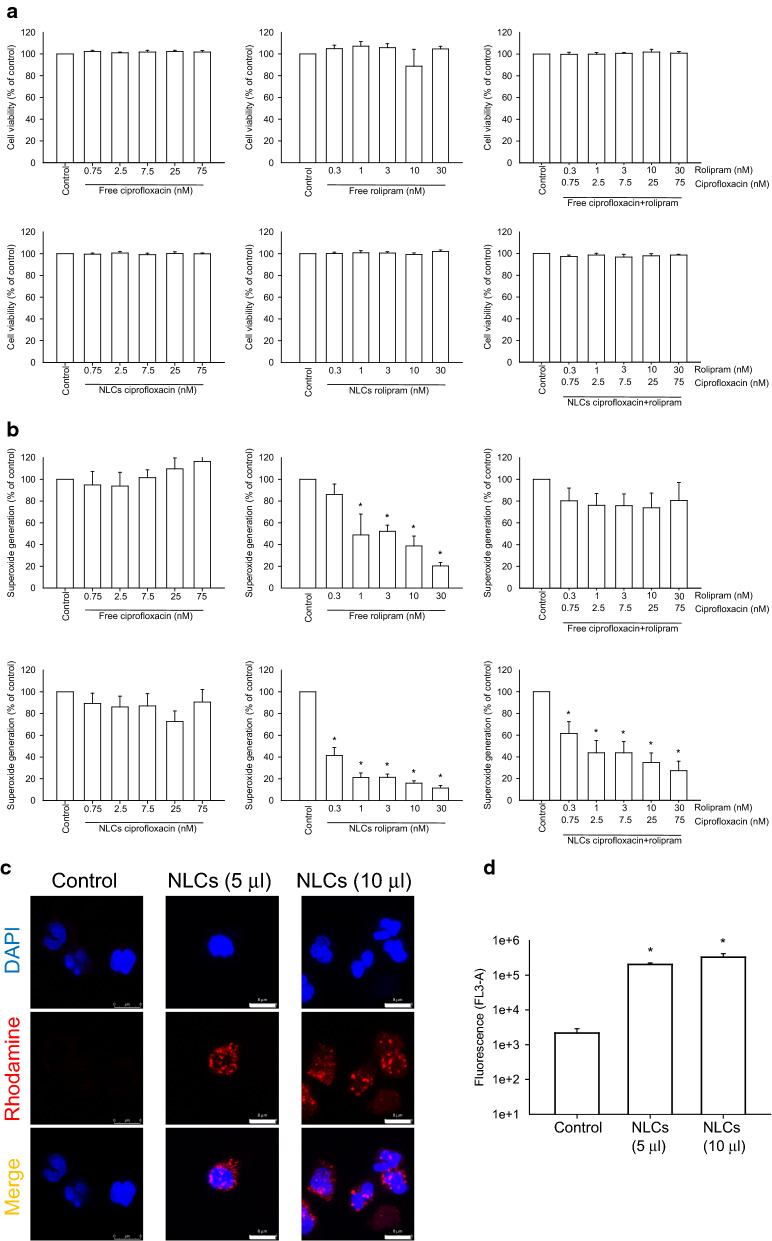
Effects of free drugs and NLCs on primary human neutrophils (6 × 10^5^ cells/ml): **a** neutrophil cytotoxicity assay by LDH determination; **b** the measurement of extracellular superoxide production; **c** the uptake of rhodamine 800-loaded NLCs by human neutrophils observed via confocal microscopy; and **d** the fluorescence intensity of rhodamine 800 in the neutrophils analyzed by flow cytometry. All data are expressed as the mean ± SEM (*n* = 3). * *p* < 0.05 as compared to control group. LDH, lactate dehydrogenase; DAPI, 4’,6-diamidino-2-phenylindole

Primary neutrophils were used as model cells to examine the anti-inflammatory activity of the NLCs and to confirm the possible activity that mitigates bacteremia-elicited acute inflammation. Prior to assessing inflammation inhibition, a cytotoxicity experiment was performed by measuring lactate dehydrogenase (LDH) leakage, and the results are shown in Fig. 2a. Various concentrations of ciprofloxacin (0.75−75 nM) and/or rolipram (0.3−30 nM) in both free and nanoparticulate forms did not exert cytotoxic effects on neutrophils. Activated neutrophils produce superoxide anions to induce oxidative stress. We observed that ciprofloxacin, in both its free and nanoparticulate forms, failed to suppress extracellular superoxide anion production (Fig. 2b). Rolipram inhibited superoxide anion production in a concentration-dependent manner, and the nanocarrier formulation (59%−88% suppression) showed greater inhibition than the free drug (17%−78% suppression). The inhibition of oxidative stress by rolipram was not a consequence of cytotoxicity since there was no LDH leakage after rolipram treatment. In fact, the incorporation of ciprofloxacin decreased the rolipram-induced superoxide inhibition. Free ciprofloxacin even completely prevented the inhibitory activity of free rolipram.

We next used confocal microscopy to visualize the uptake of the NLCs by human neutrophils. The nuclei were stained with DAPI and emitted a blue signal, as shown in Fig. 2c. Neutrophils treated with low (5 µl) and high (10 µl) amounts of NLCs emitted increased red fluorescence compared to the nontreated neutrophils. The lipid nanoparticles were predominantly located in the cytoplasm as punctate dots. The distribution of the red fluorescence was comparable between the neutrophils treated with low and high amounts of NLCs. As shown in Fig. 2d, the intracellular fluorescence, as analyzed by flow cytometry, markedly increased by a factor of 2 log after nanoparticle internalization.

### Biodistribution of NLCs in rats

**Fig. 3:**
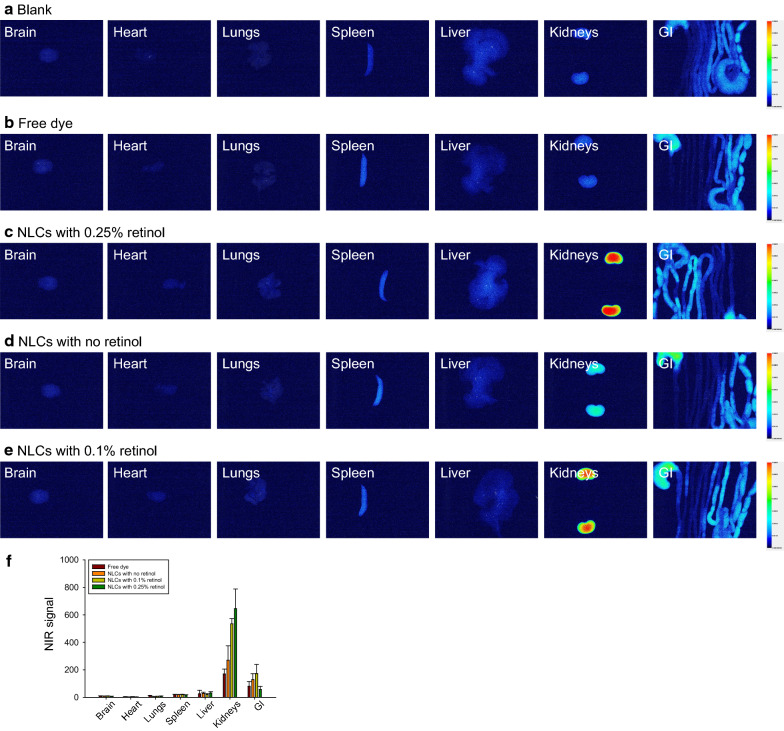
Ex vivo bioimaging of organs in the rats receiving intravenous iFlour 790-loaded NLCs: **a** the blank group (non-treated control); **b** Free iFlour 790; **c** iFlour 790-loaded NLCs containing 0.25% retinol; **d** iFlour 790-loaded NLCs containing 0% retinol; **e** iFlour 790-loaded NLCs containing 0.1% retinol; and **f** the NIR intensity of iFlour 790 in the organs. All data are expressed as the mean ± SEM (*n* = 6). GI, gastrointestinal tract; NIR, near-infrared

The distribution of NLCs after injection into the human body is important for understanding possible drug deposition in organs and for achieving effective therapy. To assess the biodistribution of NLCs in peripheral organs after intravenous injection, the lipophilic dye iFluor 790 was loaded into the nanocarriers for near-infrared (NIR) monitoring. We used healthy rats, rather than mice, as the animal model in this biodistribution experiment due to their larger organ size and lower autoNIR signal in organs, which facilitated clearer visualization. Figure 3 shows the qualitative and quantitative analyses of nanoparticle distribution by IVIS. The rats that received no treatment (blank control) exhibited negligible autoNIR signals in their organs, except for the gastrointestinal tract (Fig. 3a). The NIR signal in the organs before and after receiving free dye was comparable (Fig. 3b), indicating a rapid degradation of the dye in circulation. The intravenously injected NLCs were deposited mainly in the kidneys (Fig. 3c). The NIR signal in the other organs after nanoparticle distribution was comparable to that in the blank control group. The renal distribution of nanoparticles can be affected by retinol; therefore, we reduced the retinol concentration in the NLCs from 0.25–0% and 0.1% to visualize their biodistribution, and we found that the NIR signal was weaker (Fig. 3d and e). The quantification of the NIR intensity indicated a lower accumulation of NLCs without retinol in the kidney than that of the NLCs with retinol (Fig. 3f). After the intravenous injection of the NLCs with different percentages of retinol, there was no significant difference in the NIR intensity in other organs.

### Use of NLCs for MRSA bacteremia treatment

**Fig. 4:**
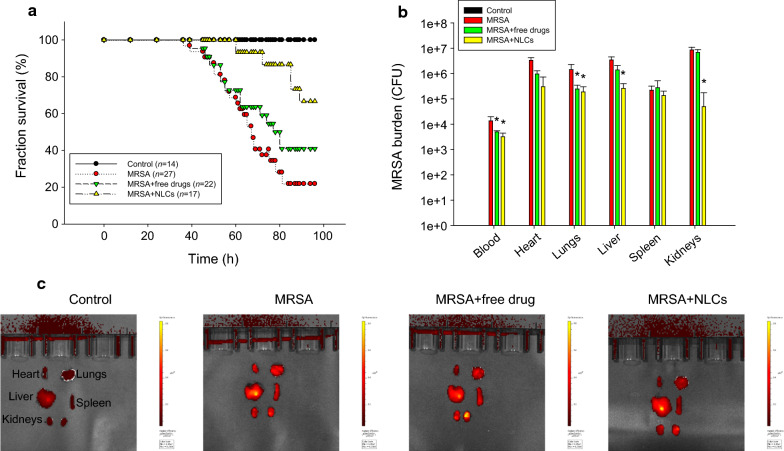
In vivo effect of free drugs and NLCs on MRSA-infected mice during 4 days: **a** the survival rate of the mice determined by Kaplan-Meier curves; **b** MRSA CFU in organs; and **c** elastase distribution in organs observed by IVIS. All data are expressed as the mean ± SEM (*n* = 14−27 for survival rate and *n* = 5 for MRSA CFU and elastase analysis). CFU, colony-forming unit; MRSA, methicillin-resistant *Staphylococcus aureus*

To evaluate the protective effects of free drugs and NLCs on MRSA-induced bacteremia in mice, we examined the survival rate of the mice four days after intravenous injection of the drug-loaded formulations. The mice infected with MRSA displayed 22% survival on day four, as shown by the Kaplan-Meier curve in Fig. 4a. Following treatment with the combination of free ciprofloxacin (2.5 mg/kg) and rolipram (1 mg/kg), 41% of the infected mice survived after four days of MRSA intervention. We found an improvement in the survival of the bacteremic mice (67%) that received NLCs containing 0.25% (w/v) retinol, suggesting significant disease remission. The surviving mice were sacrificed to quantify the MRSA accumulation in blood and peripheral organs. No MRSA was detected in the animals that did not receive bacterial injection (control) (Fig. 4b). Four days after injection, MRSA was largely deposited in the blood and organs. The MRSA colony-forming unit (CFU) count was log-transformed in this figure. Neither the free drugs nor the NLCs reduced the MRSA CFU counts in the heart and spleen. Significant inhibition of the MRSA CFU counts in the blood and lungs was observed after treating the mice with the free drugs. The NLCs caused a 1−2 log reduction in the CFU counts in the blood, lungs, liver, and kidneys compared to those in the infected mice without treatment; the greatest decrease in the CFU count was observed in the kidneys. Statistical analysis revealed that compared with the free drug control, the nanoparticles led to a significant reduction in MRSA in the liver and kidneys. To explore the anti-inflammatory activity, the excised organs were visualized by ex vivo bioimaging to assess the elastase distribution in organs. Elastase is an indicator of activated and infiltrating neutrophils. As shown in Fig. 4c, the elastase signal (bright red color) in all the organs showed an increasing trend in response to MRSA infection. The administration of the combination of ciprofloxacin and rolipram in their free forms decreased the elastase levels in the heart, but this decrease was not observed in the other organs. We observed that the elastase levels in the heart, lungs, spleen, and kidneys were reduced in the group that received the NLCs, but the NLC-treated group did not exhibited a reduction in the elastase level in the liver.

**Fig. 5:**
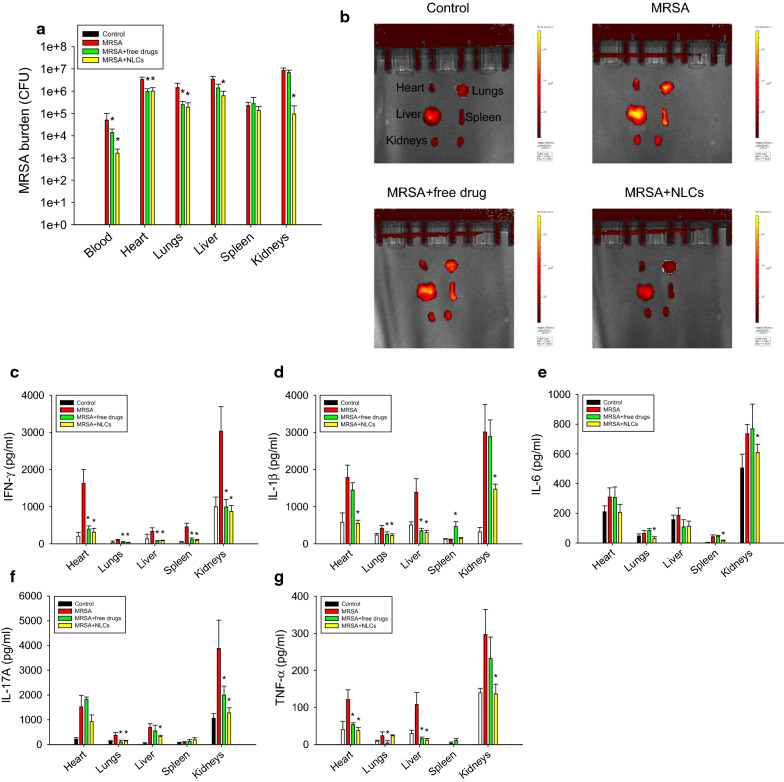
In vivo effect of free drugs and NLCs on MRSA-infected mice during 40 h: **a** MRSA CFU in organs; **b** elastase distribution in organs observed by IVIS; **c** IFN-γ expression in organs; **d** IL-1β expression in organs; **e** IL-6 expression in organs; **f** IL-17A expression in organs; **g** TNF-α expression in organs. All data are expressed as the mean ± SEM (*n* = 6 for MRSA CFU and elastase analysis). * *p* < 0.05 as compared to MRSA-infected group. CFU, colony-forming unit; IFN-γ, interferon-γ; IL-1β, interleukin-1β; IL-6, interleukin-6; IL-17A, interleukin-17A; MRSA, methicillin-resistant *Staphylococcus aureus*; TNF-α, tumor necrosis factor-α

Only a limited number of mice survived long enough to assess the MRSA burden and elastase levels; therefore, we shortened the treatment duration to 40 h for further examination. As shown in Fig. 5a, the MRSA inhibition in the heart and lungs was comparable between the free drug and NLC treatment groups. The bacterial count in the blood, liver, and kidneys was significantly reduced by the NLCs compared with the free drugs. There was a 2-log reduction in the MRSA growth in the blood and kidneys in the NLC group compared to the MRSA group without drug intervention. After the four-day treatment, neither the free drugs nor the NLCs decreased the MRSA accumulation in the spleen. The elastase distribution trends in the mice after 40 h of treatment were consistent with the results of the longer treatment (four days), as shown in Fig. 5b. The NLCs more substantially decreased the elastase levels than the free drugs in the infected mice. Next, we examined whether free drugs or NLCs suppressed proinflammatory cytokine expression in the organs involved in bacteremia. The ELISA results are shown in Fig. 5c−g. The level of IFN-γ in the MRSA-infected animals significantly increased compared with that in the healthy control animals (Fig. 5c). The administration of the combination of ciprofloxacin and rolipram in their free and nanoparticulate forms caused comparable inhibition of IFN-γ. The administration of NLCs resulted in significantly lower expression of IL-1β in the heart and kidneys (Fig. 5d). This effect was not observed in the group treated with free drugs. Treatment with free drugs and NLCs significantly inhibited the IL-1β overexpression in the lungs and liver to comparable degrees. IL-1β expression in the spleen increased in the free drug-treated mice, but not in the infected mice without drug intervention, compared to the healthy control mice. We could not explain this phenomenon with the existing knowledge. Further study is needed to delineate the possible mechanisms.

MRSA intervention did not have a significant effect on IL-6 expression in the organs, except for the spleen (Fig. 5e). Nevertheless, compared with no drug treatment, the NLCs still exhibited the capability to inhibit IL-6 expression in the lungs and spleen. This result suggests that the protein concentration of IL-17A significantly increased in all organs after MRSA challenge (Fig. 5f). The free drugs were capable of inhibiting IL-17A in the lungs and kidneys. NLC administration further reduced IL-17A production in the lungs, liver, and kidneys by 61%, 52%, and 67%, respectively. The NLCs decreased IL-17A production in the kidneys to the baseline levels observed in the controls. No IL-17A suppression was observed in the heart and spleen of infected mice after treatment with the free drugs or NLCs. TNF-α expression in all the organs increased by 2.1−3.7-fold in the MRSA-infected animals compared to that in the healthy animals (Fig. 5g). The TNF-α levels in the heart and liver were markedly reduced to baseline levels after treatment with both the free drugs and NLCs. The free drugs exerted no effect on the suppression of TNF-α overexpression in the kidneys. Nanocarrier treatment resulted in a 2-fold decrease in the TNF-α levels in the kidneys compared with those observed in the infected group.

### Histological observation

**Fig. 6:**
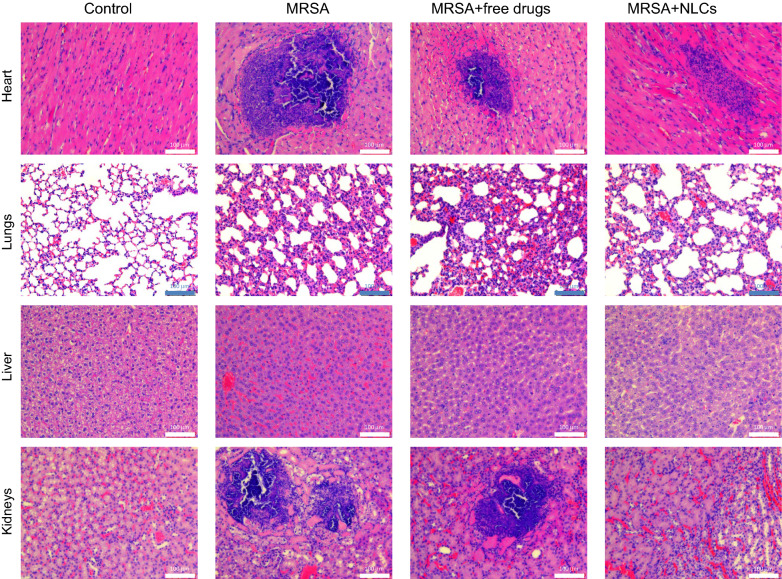
The H&E staining of the organs in the mice with or without MRSA infection. MRSA, methicillin-resistant *Staphylococcus aureus*

Forty hours after MRSA challenge, the mice were sacrificed to observe the histology of the different organs. The histology of the spleen was excluded in this experiment because of the negligible effects of free drug or nanoparticle treatment on the inhibition of MRSA and the suppression of some cytokines. The hearts of the healthy mice showed intact myocytes and cardiac muscle fibers, as shown in Fig. 6. MRSA infection resulted in a large area of cell debris with suppurative inflammation and immune cell infiltration. The area of suppurative inflammation was reduced after free drug and NLC treatment. Hematoxylin and eosin (H&E) staining of the lungs of the infected mice showed interstitial congestion with a loss of lung architecture. Edema and immune cell recruitment in the parenchyma were also observed in the MRSA-infected lungs. Moreover, interstitial thickening with congestion was worsened by free drug administration. The NLCs, however, markedly attenuated the MRSA-induced lung injury with a greater region of preserved pulmonary architecture. The hepatocytes in the liver tissues of the normal mice appeared well organized. Congestion and inflammatory cell infiltration were observed in hepatic tissues after MRSA injection. This was a typical observation of liver infection in the early stage. While congestion could be improved by the free drugs and NLCs, this improvement was limited. H&E staining revealed that the kidney structure consisted mainly of renal tubules and medulla. The renal tissues in the control mice appeared normal and regular. MRSA challenge induced cell debris accumulation and acute suppurative inflammation in the tissue. Following MRSA intervention, immune cell infiltration and tubule necrosis were also visualized. Following MRSA treatment, the kidney structure was preserved by the injection of the drugs in their free forms. Congestion and immune cell recruitment were reduced by the NLCs compared to those observed in the infected group.

**Fig. 7:**
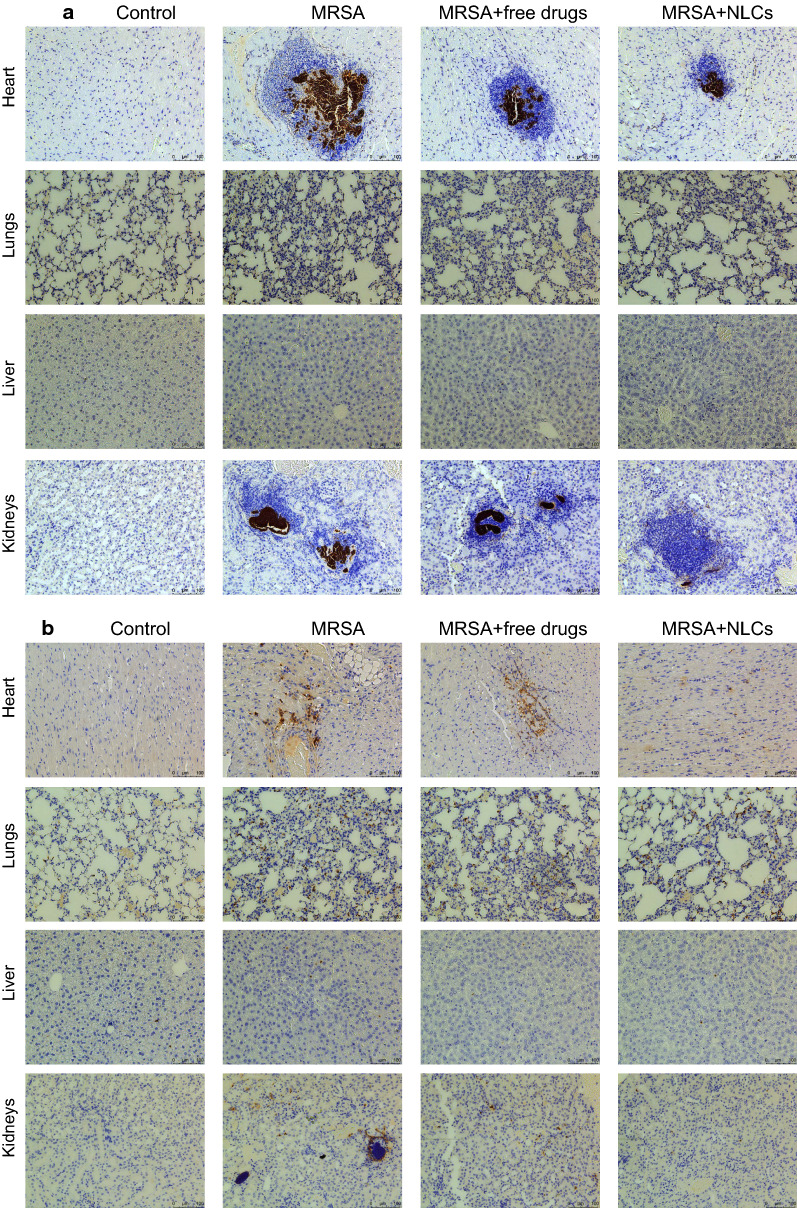
The histology of the organs in the mice with or without MRSA infection: **a** MRSA distribution determined by *S. aureus* Rosenbach antibody; and **b** neutrophil distribution determined by recombinant anti-Ly6G antibody. MRSA, methicillin-resistant *Staphylococcus aureus*

The MRSA distribution in the organs was examined using *S. aureus* Rosenbach antibody immunohistochemistry, as shown in Fig. 7a. There were bacterial clusters in the hearts of the MRSA-treated mice. These clusters were limited by the free and nanoparticulate forms of the drugs. The MRSA distribution in the lungs was also inhibited by the free drugs and NLCs. However, the MRSA distribution in the liver could not be clearly observed in all the groups. This could be due to the minimal aggregation of MRSA in the liver, which makes it difficult to recognize abscesses in the tissue. The MRSA bacterial clusters in the kidneys were reduced by the NLCs but not by the free forms of the drugs. Ly6G is a biomarker of neutrophil migration into organs that is used to characterize the features of sepsis. We visualized neutrophil accumulation in the heart 40 h after MRSA injection, as shown in Fig. 7b. Neutrophil diffusion was attenuated by the administration of the drugs, and the NLCs demonstrated better performance. On the other hand, the neutrophil infiltration in the lungs and liver caused by MRSA was reduced by the drugs in either their free forms or nanoparticulate forms; however, this reduction was not very significant. Neutrophils expressing Ly6G were clearly detected in the kidneys after MRSA infection. The number of neutrophils was markedly reduced after nanocarrier injection, indicating an efficient alleviation of infection-associated inflammation.

## Discussion

Early initiation of the inhibition of bacterial growth and inflammation is crucial for the treatment of bacteremia and sepsis. The current therapy for mitigating bacteremia-induced sepsis is unsatisfactory for achieving a successful outcome. Improving drug delivery systems is one of the strategies for enhancing therapeutic efficiency against bacteremia. In our study, we carried out thorough analyses to assess the capability of treating bacteremia with NLCs incorporating both antibacterial and anti-inflammatory drugs. We found that NLCs were more effective in eradicating MRSA than free drugs (Fig. 1). A similar trend was observed for inhibiting superoxide anion production by activated neutrophils (Fig. 2b). Our results demonstrate a large accumulation of intravenously injected NLCs in the kidneys (Fig. 3). This accumulation was beneficial for alleviating the renal dysfunction caused by bacteremia. Intravenous delivery of dual drug-loaded nanocarriers prevented the worsening of sepsis in mice and elevated the survival rate (Fig. 4a). Nanomedicine has been widely used due to its success in eradicating drug-resistant bacteria in vitro or in vivo [27–32]. We focused on the effect of nanocarriers encapsulating two drugs on the treatment of the bacteremia induced by MRSA. We also found that retinol incorporation in NLCs was beneficial for renal targeting in order to treat kidney injury.

The application of high-pressure homogenization and sonication was used to obtain NLCs of small particle size (Table 1). Particle diameters of 10−200 nm are most relevant for exerting biological effects [33]. For lipid-based nanoparticles, a PDI of ≤ 0.3 is considered to be acceptable [34]. Our nanosystems showed a PDI of 0.42, and they can be considered highly polydisperse [35]. Nevertheless, the particle diameter was maintained at low size (171 nm) with a similar PDI in the three batches (0.42 ± 0.04). We therefore concluded that the quality control of the nanocarriers was adequate. The nanocarriers prepared in the present study fit this criterion. Cationic nanoparticles are associated with hemolysis after intravenous administration [36]; therefore, we designed nanoparticles with a negative surface charge to prevent hemolysis. We incorporated deoxycholic acid into the nanoformulations to obtain a negative charge of –39 mV. This value was advantageous in showing sufficient storage stability because a zeta potential of > 30 or <–30 mV indicates good electrostatic stabilization [37]. The high rate of rolipram encapsulation in the NLCs was attributed to the high lipophilicity of this compound, which allowed it to be retained in the lipid cores of the NLCs. Although ciprofloxacin is less lipophilic than rolipram, the encapsulation percentage of this drug reached nearly 90%. The mixture of lipids in solid and liquid forms in the cores of NLCs leads to imperfection in the matrix, which allows greater space for loading both lipophilic and hydrophilic chemicals [38].

Ciprofloxacin, but not rolipram, demonstrated anti-MRSA activity. The degree of MRSA suppression by ciprofloxacin was significantly reduced after combined treatment with rolipram in either the free or nanoparticulate forms (Table 2). A previous study [39] verified the possibility that ciprofloxacin could exhibit reduced antibacterial activity in the presence of PDE4 inhibitors. In addition to their anti-inflammatory properties, PDE inhibitors have other bioactivities, such as antioxidative activity and protective activity against oxidative stress. Given the antibacterial mechanisms of ciprofloxacin, namely, the induction of reactive oxygen species to kill microbes, PDE inhibitors may restrict the antibacterial potency of ciprofloxacin. A possible drug-drug interaction between PDE4 inhibitors and ciprofloxacin was also observed in a previous investigation [40]. The combined treatment also exhibited a similar trend in its anti-inflammatory activity against activated neutrophils (Fig. 2b). The production of oxidative stress by ciprofloxacin may be the reason for the reduced ability of rolipram to inhibit superoxide anion production. The inclusion of ciprofloxacin in the nanoparticles inhibited MRSA growth compared to the free drugs (Fig. 1). Sustained and slow release of the drug from nanocarriers maintains drug retention near bacteria over a long period [9]. The surface of NLCs containing phosphatidylcholine and poloxamer 188 can easily fuse with the bacterial cell wall [41], resulting in the accumulation of antibacterial agents in large amounts in microbes.

Neutrophils are the major phagocytic cells that act as first-line responders in the fight against pathogenic microbes. Once uncontrolled activation of neutrophils occurs at the infected site, organs are possibly damaged. Neutrophils are recognized as essential players in acute inflammation, such as the inflammation elicited by bacteremia, sepsis, and traumatic hemorrhage [42]. Neutrophil stimulation is affected by the intracellular cAMP level, which can be regulated by PDE4 inhibition. Rolipram is a selective PDE4 inhibitor that reduces the number and activation of infiltrating neutrophils in inflammatory regions [43]. For intracellular drug delivery to inhibit neutrophil activation, nanocarriers must be internalized by neutrophils, and then, the drug is released. Our results demonstrated the easy ingestion of NLCs by neutrophils (Fig. 2c). A high zeta potential level, either positive or negative, has been established as a vital factor for assisting phagocytosis by neutrophils [44]. Soft nanoparticles can be deformed by cells during phagocytosis. This uptake is energetically less favorable [45]. Lipid-based nanoparticles can be classified as soft but not rigid structures that facilitate easy internalization by cells [46]. Neutrophil influx into peripheral organs mediates sepsis through superoxide generation. This oxidative stress induced by stimulated neutrophils is inactivated by the internalization of rolipram-loaded NLCs by neutrophils, which results in greater superoxide inhibition than drugs in free form (Fig. 2b).

An important feature for the treatment of bacteremia and sepsis is the appropriate penetration of drugs into tissues to limit organ damage. The therapeutic outcome can be improved when the drugs are delivered to the target organs. The biodistribution of intravenously injected NLCs showed the easy delivery of the nanoparticles to the kidneys (Fig. 3). An earlier study [12] also suggested higher deposition of NLCs in the kidneys than in other organs. Our NLCs possessed a size (150−180 nm) similar to that of the NLCs that were previously developed. The biodistribution results revealed a higher accumulation of NLCs with a higher concentration of retinol in the kidneys (Fig. 3). In the bloodstream, retinol is bound to specific retinol-binding proteins (RBPs), especially RBP4. RBPs play a principal role in promoting retinol transport in the body [47]. RBP4 is a plasma protein mainly secreted from the liver to transport retinol to peripheral organs via the bloodstream. The kidneys possess abundant RBP receptors with high retinol-binding activity [48]. In rats, approximately 50% of plasma retinol turnover is associated with the kidneys [49]. This may be the reason for the increased kidney deposition of the NLCs with high levels of incorporated retinol. The level of RBP4 also increases during renal dysfunction but not during hepatic dysfunction [50]. Although the liver is also a main target of retinol transport, we observed minimal NLC deposition in this organ (Fig. 3). This result is quite different from the lipid-based nanoparticles prepared in our previous study [51], which demonstrated a large accumulation of retinol-loaded nanoparticles in the liver. The encapsulation percentage of retinol in the nanoparticles in the previous study was 0.02%, which was much lower than that in the present study (0.25%). It is difficult to compare the different nanocarriers since each nanosystem has its own intrinsic nature. In addition to the incorporation of materials, other factors, such as size, surface charge, and loaded drugs, also influence nanoparticle biodistribution. The biodistribution is nanocarrier-dependent and cannot be applied universally to all nanoformulations.

Once nanoparticles are administered into the blood, plasma proteins adsorb onto the particulate surface for opsonization. Opsonized nanoparticles are recognized by the mononuclear phagocyte system to be removed from circulation into the liver and spleen [52]. Nanoparticles with larger lipophilic surfaces tend to adsorb more proteins in circulation. The enhanced hydrophilic nature of the NLC surface can prevent uptake by the liver and spleen and thus prolong the circulation time [53]. The attachment of hydrophilic polymers, including polyethylene glycol, poloxamer, and chitosan, is effective for avoiding hepatic clearance [54]. Cationic nanoparticles show a high affinity for plasma proteins and parenchymal cells, leading to a broad distribution in the liver [55]. In our NLCs, the presence of poloxamers and a negative charge on their surfaces contributed to their low accumulation in the liver and spleen (Fig. 3). The nanoparticulate diameter is a key parameter for controlling their biodistribution. Nanocarriers larger than 200 nm are rapidly delivered to the liver and spleen because of complement activation [56, 57]. A previous study [58] also suggested that nanoparticles with sizes of 50−100 nm and < 50 nm are largely distributed in the hepatic parenchyma and spleen, respectively. According to this evidence, the size of our NLCs might have enabled their escape to enter the liver and spleen. The kidneys have fenestrated capillaries that facilitate the entrance of nanoparticles [55]; however, nanoparticles with a size > 6 nm cannot be removed from the kidneys [54]. Dual drug-loaded NLCs could penetrate but could not flow out of the kidneys, causing their accumulation in renal tissues. The uptake of NLCs by the brain is negligible, which could be due to the incorporation of poloxamer 188 to generate hydrophilic and steric surfaces. These surfaces are not conducive to delivery to the brain [12]. Limited brain targeting is favorable for rolipram delivery because this compound causes neurological disorders as side effects when it enters the brain.

In severe sepsis, the development of acute cardiac and renal dysfunction is commonly observed. *S. aureus* bacteremia has emerged as the most common cause of infective endocarditis [59]. Our results indicated decreased MRSA burden and elastase distribution in the heart after treatment with the combination of ciprofloxacin and rolipram (Fig. 5a and b). Moreover, NLCs exerted superior effects compared with free drugs. A previous study [60] demonstrated that ciprofloxacin is largely distributed in the cardiac cavity after injection. This observation explains the effectiveness of free ciprofloxacin in eradicating MRSA in the heart. Acute kidney injury is a major complication that arises due to sepsis. NLCs were helpful in inhibiting MRSA growth and elastase upregulation in the kidneys (Fig. 5a and b). This effect was not observed in the group treated with the free drugs, although PDE4 inhibition has been proven to attenuate acute renal failure in sepsis and endotoxemia [24, 61].

NLCs showed high efficiency for encapsulating ciprofloxacin and rolipram. Drugs encapsulated in nanoparticles can be protected from enzymatic attack to facilitate delivery to renal tissue. Ex vivo bioimaging in this study confirmed the large distribution of NLCs in the kidneys (Fig. 3). NLCs noticeably decreased the bacterial load and neutrophil recruitment in organs other than the kidneys, although the nanoparticle biodistribution in these organs was low. A large number of bacteria and neutrophils are present in bacteremic blood. It is suggested that NLCs inhibit MRSA growth and inflammation in circulation in the early stage of infection, followed by limited bacterial and neutrophil transport to peripheral organs. Ciprofloxacin molecules are rapidly cleared from circulation with 20%−40% protein binding [61, 62]. The adsorption of plasma proteins to the nanoparticulate surface is generally low to evade degradation. NLCs with a size of < 200 nm, a poloxamer 188-incorporated surface and negative zeta potential can be retained in the bloodstream for an extended period of time [63]. Poloxamer 188 increases the hydrophilicity of nanoparticulate surfaces, resulting in diminished macrophage recognition and phagocytosis. The crystalline solid lipids present in NLCs cause slower degradation of the lipid matrix to produce long-term circulation formulations [12]. Nanoparticle uptake by neutrophils might occur in circulation to arrest neutrophil activation and subsequent migration to organs.

The mortality associated with sepsis depends on the dysregulated host cytokine storm. Cytokines are inflammatory mediators that trigger inflammatory pathology and subsequent organ damage. The cytokines IL-1β, IL-6, and TNF-α mediate the immunopathological features of sepsis and are released by neutrophils to exacerbate acute inflammation [64]. IL-6 is an early biomarker in bacteremia that is used to evaluate the inflammatory response [6, 65]. TNF-α is produced in response to acute sepsis. IL-1β and IL-6 enhance TNF-α expression to stimulate neutrophil recruitment. Activated neutrophils are the major inflammatory cells that express IL-17A, which can be used as a predictor of *S. aureus* bacteremia [66]. The organs of MRSA-infected rats showed increased levels of these cytokines (Fig. 5c to g). Our nanocarriers inhibited cytokine expression, suggesting their capacity to inhibit inflammation. The NLCs containing antibiotics and PDE4 inhibitors could inhibit MRSA growth and neutrophil activation to limit the cytokine storm caused by the bacteremia-induced inflammatory response. The acute inflammation induced by dysregulated cytokines during sepsis leads to vasodilation and apoptosis in different tissues [23]. After MRSA challenge, we observed congestion and necrosis in the heart, lungs, and kidneys (Fig. 6). The increased permeability of blood vessels allowed neutrophils to migrate from the blood to peripheral organs. Histological analysis of Ly6G showed neutrophil accumulation in the organs (Fig. 7b), while NLCs inhibited the entrance of neutrophils into organs and thus prevented organ disruption. The organ failure caused by the host response to infection is a predominant mechanism of sepsis-related death. The mortality associated with bacteremia is dependent on cytokine overexpression, and attenuated cytokine release is a prerequisite for reducing mortality [61]. In this study, the mortality rate notably increased in the MRSA-infected mice (Fig. 4a), suggesting successful creation of the bacteremia model. Rolipram was found to be useful in improving cardiac and renal function during sepsis, thus promoting the survival of the animals [24]. Our data confirmed the role of rolipram in improving survival. This amelioration was further enhanced by the encapsulation of rolipram in lipid-based nanocarriers. The NLCs suppressed the release of proinflammatory cytokines in bacteremic mice, decreased the MRSA load, and increased survival.

There were some limitations in our study. The bacteremia model established in this study and its clinical relevance are still unclear. Treatment with drugs prior to the induction of bacteremia led to an effective and stable plasma drug concentration. However, in clinical practice, it is difficult to administer drugs before bacteremia onset; thus, our method of using NLCs to treat bacteremia could be used for prevention but not for therapy with rigorous definitions. The experimental periods for the in vivo study (40 h and 4 days) were too short to offer more information regarding the long-term outcome and augment the importance of our work. Additional basic studies are required to verify any potential effect of NLCs on bacteremia. The combination of free ciprofloxacin and rolipram might cause interactions that would lower their antimicrobial and anti-inflammatory activities, but the detailed mechanisms are not completely understood. Further investigation is required to explore the inherent mechanisms.

## Conclusions

The present work demonstrated the successful preparation of dual drug-loaded NLCs to enhance the efficiency of bacteremia treatment. The encapsulation of ciprofloxacin and rolipram into lipid-based nanoparticles was nearly complete. In vitro evaluation revealed superior antibacterial and anti-inflammatory potencies of the NLCs compared to the combination of free drugs using MRSA and neutrophils as models, respectively. Intravenously injected NLCs were deposited primarily in the kidneys due to the incorporation of retinol in the nanoparticles. Treatment with NLCs encapsulating both ciprofloxacin and rolipram improved bacterial clearance, inhibited elastase expression, and prevented organ damage in rats infected with MRSA. The nanocarriers were found to suppress the cytokine overexpression in peripheral organs and improve the survival rate of rats with bacteremia-induced sepsis. The intravenous nanocarriers possibly mitigated MRSA growth and suppressed neutrophil activation in circulation, followed by minimizing MRSA and neutrophil transport to organs. The combination of anti-MRSA and anti-inflammatory nanocarriers relieved the acute inflammation caused by MRSA infection and thus showed potential application for bacteremia treatment.

## Methods

### Preparation of NLCs

The NLCs were fabricated by the homogenization-sonication method [41]. The aqueous and lipid phases were prepared separately. Three hundred fifty milligrams of Poloxamer 188 and 100 mg of deoxycholic acid were dissolved in water (8.89 ml). The lipid phase consisted of squalene (400 mg), hexadecyl palmitate (100 mg), soybean phosphatidylcholine (Phospholipon 80 H, 150 mg), retinol (25 mg), ciprofloxacin (5 mg), and rolipram (2 mg). Both phases were heated at 85°C for 15 min. Then, the aqueous phase was dispersed into the lipid phase with high-shear homogenization at 12,000 rpm for 20 min. The mixture was further sonicated using a probe-type sonicator at 35 W for 15 min. The final weight of the NLCs was 10 g.

### Estimation of the particle diameter and zeta potential

The average diameter and zeta potential of the NLCs were evaluated by a Nano ZS90 analyzer (Malvern). All the samples were diluted 100-fold with water. Each sample was analyzed in triplicate.

### The encapsulation efficiency of the drugs

The encapsulation percentage of ciprofloxacin and rolipram was calculated by using the ultracentrifugation method to separate the encapsulated drugs from the free forms. The NLCs were centrifuged at 48,000x *g* and 4°C for 40 min. The free drugs in the supernatant and encapsulated drugs in the precipitate were analyzed by high-performance liquid chromatography (HPLC), whose setups for ciprofloxacin and rolipram have been previously described [67, 68].

### Drug release from the nanoparticles

The drug release of the ciprofloxacin- and rolipram-loaded NLCs was studied by the dialysis bag diffusion method. The drug-loaded control vehicle or NLCs (5 ml) were dispersed in a dialysis bag, and the dialysis bag (Cellu-Sep T2 with an MW cutoff of 6000–8000 Da) was then incubated in a beaker containing 100 ml of pH 7.4 phosphate buffer. The free control was drugs dissolved in 35% ethanol/pH 7.4 buffer. The beaker was placed on a magnetic stirrer with a stirring rate of 100 rpm, and the temperature of the assembly was maintained at 37 °C. Samples (1 ml) were collected at definite time intervals and replaced with equal amounts of fresh buffer. After suitable dilutions, the samples were analyzed using HPLC.

### Minimum bactericidal concentration (MBC)

MRSA (ATCC33591) was purchased from the American Type Culture Collection. The MRSA suspension was diluted in tryptone soy broth to obtain a concentration of OD_600_ = 0.01. The TSB plate was incubated with different concentrations (0−31.25 µg/ml) of ciprofloxacin with or without rolipram either in their free or nanoparticulate forms at 37°C for 20 h. The CFU was then counted. The MBC was defined as the lowest ciprofloxacin concentration required to kill ≥ 99.9% of MRSA (ATCC33591).

### Time‐response MRSA growth inhibition

The inhibition of MRSA growth by the drugs in their free or nanoparticulate form within 24 h was measured in 96-well plates. Ciprofloxacin (0.5 or 1 µg/ml), rolipram (0.2 or 0.4 µg/ml), or their combination was incubated with MRSA (OD_600_ = 0.01) at 35°C for 24 h. The absorbance of each well was measured at 600 nm to detect MRSA growth in real-time.

### Isolation of human neutrophils

Neutrophils from healthy donors between the ages of 20 − 30 years old were isolated by using a protocol approved by the Institutional Review Board at Chang Gung Memorial Hospital. Written informed consent was obtained from every subject. The neutrophils were purified by sedimentation prior to centrifugation in a Ficoll Hypaque gradient and the hypotonic lysis of erythrocytes [69].

### Neutrophil viability

Lactate dehydrogenase (LDH) is an indicator of cellular membrane leakage and cell viability. The commercial kit CytoTox 96 (Promega) was used to estimate the LDH levels. The detailed protocol was previously described [70].

### Superoxide anion production by neutrophils

Superoxide anion release from primary neutrophils was assessed using Ferricytochrome *c* [71]. Briefly, neutrophils (6 × 10^5^ cells/ml) were incubated with ferricytochrome *c* (0.5 mg/ml) and CaCl_2_ (1 mM) at 37°C. Neutrophils were treated with ciprofloxacin (0.75−75 nM), rolipram (0.3−30 nM), or their combination in free or nanoparticulate form for 10 min. Then, the neutrophils were activated by formyl-methionyl-leucyl phenylalanine (fMLF, 0.1 µM) and cytochalasin B (1 µg/ml). Ferricytochrome *c* reduction was monitored by measuring the absorbance at 550 nm.

### Nanoparticle uptake by neutrophils

NLCs were labeled with 0.1 mg/ml rhodamine 800 dye to observe their ingestion by neutrophils. Neutrophils (1 × 10^7^ cells/ml) were incubated with NLCs (5 or 10 µl) at 37°C for 5 min, and the reaction was stopped by adding Hank’s balanced salt solution at 4°C. The neutrophils were imaged using confocal microscopy. The fluorescence intensity of the rhodamine-labeled NLCs was also quantified by flow cytometry.

### Animals

Male Sprague-Dawley rats (200−300 g) and Balb/c mice (20−25 g) were purchased from Lasco Biotechnology (Taipei, Taiwan). All the animals were treated in accordance with protocols approved by the Institute of Animal Care and the Use Committee of Chang Gung University.

### Biodistribution of NLCs in rats

Near-infrared (NIR) dye (iFluor 790; 0.08%) was incorporated into the NLCs to monitor the biodistribution of intravenously injected NLCs. The rats were anesthetized with Zoletil 50 (30 mg/kg) and xylazine (6 mg/kg). The NLCs (0.8 ml/kg) were administered via the tail vein. The rats were sacrificed after 2 h. The organs were harvested to detect the NIR signals with an *in vivo* imaging system (IVIS, Pearl Impulse Imaging System, Li-Cor). The NIR signal intensity was quantified by Pearl Impulse software.

### Bacteremia model induced by MRSA in mice

A mouse model of bacteremia was induced according to a previous study with some modifications [72]. The mice were randomly assigned into four groups: (i) uninfected control receiving PBS, (ii) MRSA infection, (iii) MRSA infection with intravenous injection of two drugs in 10% ethanol, and (iv) MRSA infection with intravenous injection of dual drug-loaded NLCs. Bacteremia was induced by intravenous injection of MRSA (1 × 10^7^ CFU). The mice were administered NLCs or 10% ethanol containing ciprofloxacin (2.5 mg/kg) and rolipram (1 mg/kg) 24 h before injection with MRSA. Mortality was monitored and recorded over four days with a camera. The mice were sacrificed at 40 h and 4 d postinfection to estimate the MRSA CFU counts in the blood and peripheral organs.

### Elastase in organs

The neutrophil elastase 680 FAST imaging agent (PerkinElmer) was used to observe the levels of neutrophil elastase in vivo. Four hours after the intravenous injection of the imaging agent (100 µl), the animals were sacrificed to harvest the organs. The harvested organs were observed and photographed using a fluorescence IVIS system (Lumina LT Series III, PerkinElmer).

### Cytokine expression in organs

Samples from the organs of mice were used to measure the levels of cytokines, including IFN-γ, IL-1β, IL-6, IL-17A, and TNF-α, using commercial kits (BioLegend), following the manufacturer’s instructions.

### Histological observation

Specimens from different organs were immersed in 10% formaldehyde, embedded in paraffin, and then sliced into 5-µm-thick sections for H&E staining. We also examined MRSA and Ly6G in the organs by immunohistochemistry. The organ sections were incubated with the relevant antibodies (*S. aureus* Rosenbach antibody for MRSA and recombinant anti-Ly6G antibody for neutrophils) for 1 h and then incubated with biotinylated donkey anti-rabbit IgG for 20 min. The sections were observed by optical microscopy.

### Statistical analysis

The data shown in this work are presented the mean and the standard error of the mean. Significant differences between different groups were examined by the Kruskal-Wallis method. Individual differences were evaluated post hoc by Dunn’s test. Significance was demonstrated by *p* < 0.05.

## Supplementary Information

**Additional file 1: Figure S1.** The release kinetics of ciprofloxacin androlipram from free control and NLCs.

## Data Availability

The datasets used and/or analysed during the current study are available from the corresponding author on reasonable request.
