# Supplementary figures and images for: Multifunctional lipid-based nanocarriers with antibacterial and anti‐inflammatory activities for treating MRSA bacteremia in mice
Source: J Nanobiotechnology. 2021 Feb 15;19:48. doi: 10.1186/s12951-021-00789-5 (PMC7885212; doi:10.1186/s12951-021-00789-5)

## Slide 1
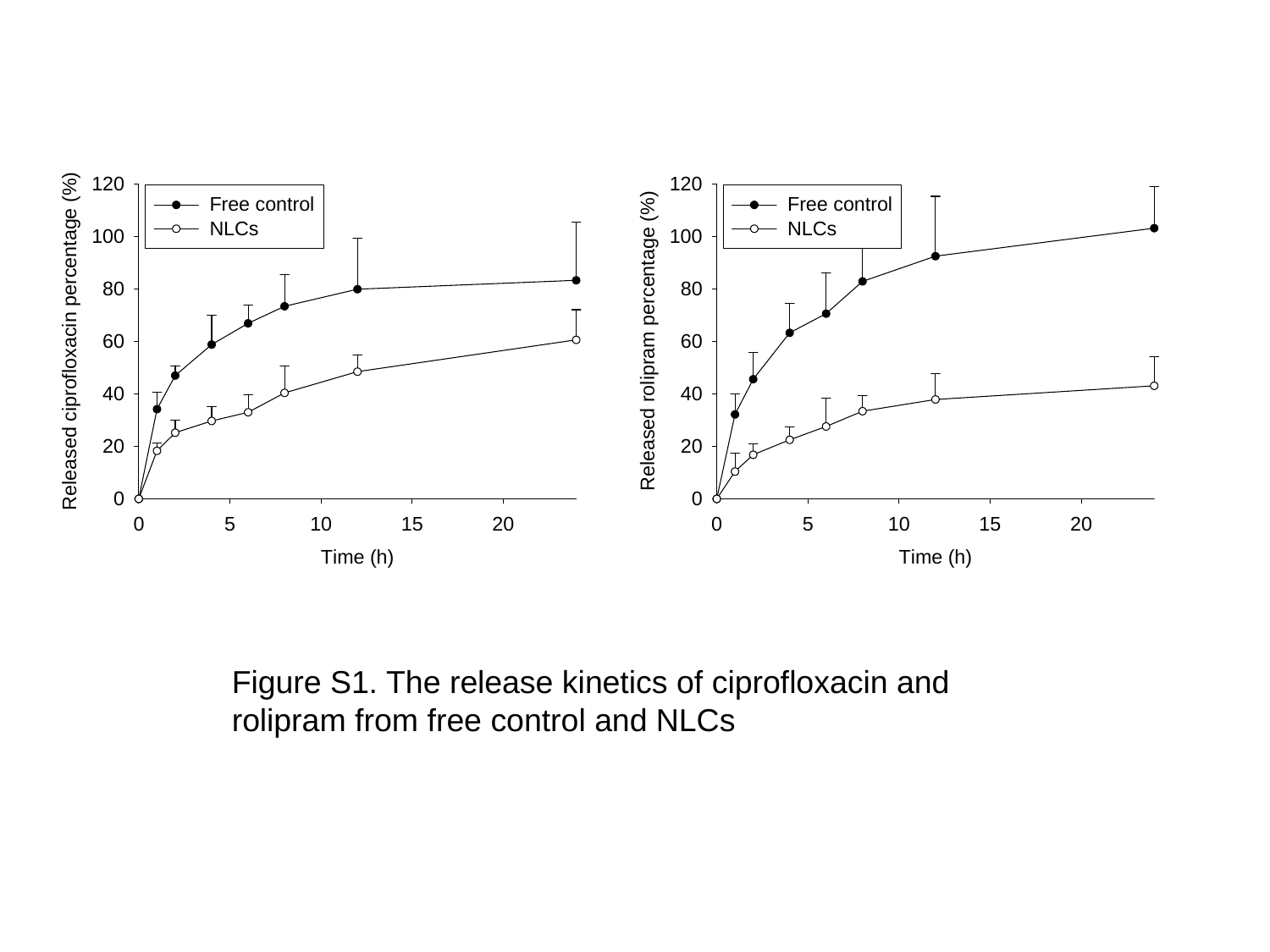

Figure S1. The release kinetics of ciprofloxacin and
rolipram from free control and NLCs

Supplement: Supplementary file 1 — Additional file 1: Figure S1. The release kinetics of ciprofloxacin androlipram from free control and NLCs. [file 12951_2021_789_MOESM1_ESM.pptx]
